# The Role of RNA Splicing in Liver Function and Disease: A Focus on Metabolic Dysfunction-Associated Steatotic Liver Disease

**DOI:** 10.3390/genes15091181

**Published:** 2024-09-08

**Authors:** Dorota Kaminska

**Affiliations:** Department of Medicine, Division of Cardiology, David Geffen School of Medicine, University of California Los Angeles, Los Angeles, CA 90095, USA; dkaminska@ucla.edu

**Keywords:** alternative splicing, Metabolic Dysfunction-Associated Steatotic Liver Disease, Metabolic Dysfunction-Associated Steatohepatitis, MASLD, MASH

## Abstract

RNA splicing is an essential post-transcriptional mechanism that facilitates the excision of introns and the connection of exons to produce mature mRNA, which is essential for gene expression and proteomic diversity. In the liver, precise splicing regulation is critical for maintaining metabolic balance, detoxification, and protein synthesis. This review explores the mechanisms of RNA splicing and the role of splicing factors, particularly in the context of Metabolic Dysfunction-Associated Steatotic Liver Disease (MASLD). This review also highlights how RNA splicing dysregulation can lead to aberrant splicing and impact the progression of liver diseases such as MASLD, with a particular focus on Metabolic Dysfunction-Associated Steatohepatitis (MASH), which represents the advanced stage of MASLD. Recent advances in the clinical application of antisense oligonucleotides (ASOs) to correct splicing errors offer promising therapeutic strategies for restoring normal liver function. Additionally, the dysregulation of splicing observed in liver diseases may serve as a potential diagnostic marker, offering new opportunities for early identification of individuals more susceptible to disease progression. This review provides insights into the molecular mechanisms that govern splicing regulation in the liver, with a particular emphasis on MASLD, and discusses potential therapeutic approaches targeting RNA splicing to treat MASLD and related metabolic disorders.

## 1. Introduction

The liver is a vital organ responsible for maintaining the overall body’s metabolic balance. Key functions include managing bile acids and cholesterol, neutralizing harmful substances, producing and storing glucose, processing fats, regulating hormones, and secretion of liver-generated proteins to circulation. Hepatocytes, the primary liver cells comprising 80% of its mass and 60% of its cellular makeup, are primarily responsible for these activities [1]. To execute these intricate functions, hepatic cells require the expression of a wide range of genes precisely regulated by a complex interplay of transcription factors and RNA splicing modulators. While the transcriptional control of gene expression in the liver has been extensively studied for decades, the mechanisms governing liver-specific alternative splicing remain relatively unexplored.

Alternative splicing is an essential mechanism that regulates gene expression, providing a means to generate multiple mRNA transcripts from a single gene, thus enhancing the variety of the proteome. This process is vital for the development and function of complex organisms. The importance of alternative splicing in biology was highlighted by the Nobel Prize in Physiology or Medicine awarded in 1993 to Richard J. Roberts and Phillip A. Sharp, who discovered that genes are frequently interrupted by non-coding regions known as introns [2,3]. Their pioneering work revealed that pre-mRNA can undergo alternative splicing to generate multiple mRNA variants, a discovery that has had profound implications for our understanding of genetic complexity and regulation.

Alternative splicing has been increasingly implicated in the pathogenesis of complex diseases [4], including metabolic diseases such as obesity (reviewed elsewhere [5,6]) and Metabolic Dysfunction-Associated Steatotic Liver Disease (MASLD) [7,8,9,10,11]. 

Given the critical role of splicing in regulating gene expression, therapeutic strategies targeting aberrant splicing or the splicing factors that govern this process offer promising avenues for treating liver diseases. By specifically targeting the dysregulated splicing mechanisms associated with MASLD and related conditions, therapies can offer a novel approach to mitigating disease progression and improving patient outcomes. This review will explore the potential of these therapeutic strategies, providing an overview of their mechanisms of action and future directions in the treatment of MASLD.

## 2. Different Types of Splicing

**Figure 1:**
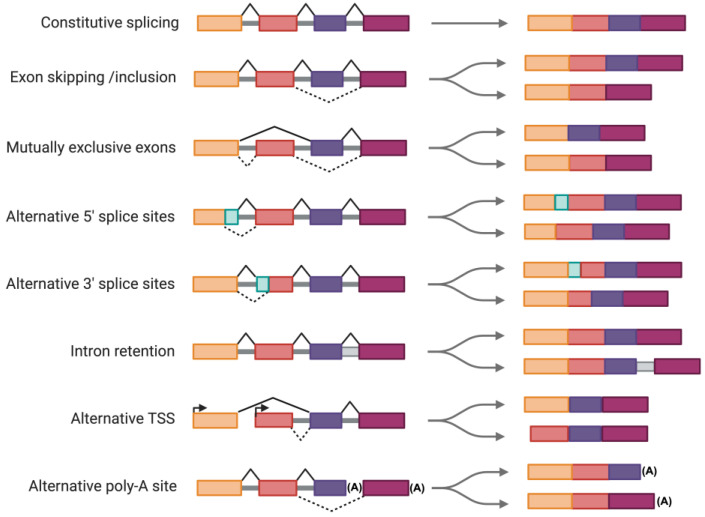
Various forms of alternative splicing. In this diagram, exons are shown as colored boxes, introns are depicted with solid gray lines, and black solid and dashed lines indicate possible splicing variations. Transcription start sites (TSS) are indicated by arrows, and polyadenylation sites are represented by the symbol (A). The figure was created using BioRender (www.biorender.com, accessed on 4 September 2024).

Splicing is a fundamental gene expression mechanism in eukaryotic cells, where non-coding introns are excised from pre-mRNA, and coding exons are connected to generate mature mRNA. There are two main types of splicing, each contributing to the diversity and regulation of the transcriptome [12]. Constitutive splicing is the most straightforward form of splicing, where exons are sequentially joined in the same order as they occur in the gene, resulting in the production of a single mRNA transcript. In contrast, alternative splicing enables the production of diverse mRNA isoforms from a single gene by including or excluding certain exons or by utilizing alternative splice sites. This type of splicing can result in different protein isoforms with varying functions, increasing proteomic complexity. Exon skipping is a common form of alternative splicing where specific exons are omitted from the final mRNA. Mutually exclusive exons are another form where only one of two exons is included in the mRNA, but not both. Alternative 5′ and 3′ splice sites involve the use of different donor or acceptor sites within an exon, changing the exon’s boundaries. Finally, intron retention, where introns are retained in the mature mRNA, can regulate gene expression by affecting mRNA stability, transport, or translation. Alternative transcription start sites (TSS) and alternative polyadenylation sites represent additional forms of splicing that contribute to transcript diversity (Figure 1). Each of these types is tightly regulated by a complex interplay of *cis*-regulatory sequences and *trans*-acting splicing factors, ensuring the precise control of gene expression necessary for cellular function and adaptation through an efficient production of diverse protein isoforms. Circular RNA (circRNA) represents a unique category of RNA molecules that are generated through a special form of alternative splicing known as reverse splicing or back-splicing [13]. Unlike linear mRNA, which has distinct 5′ and 3′ ends, circRNAs form a covalently closed continuous loop, lacking these terminal ends. This unique structure arises when a 5′ splice site of a downstream exon is connected to a 3′ site of an upstream exon, looping out the intervening exons. CircRNAs are increasingly recognized for their roles in gene regulation and are abundant in various tissues, including the liver [14]. The role of back-splicing and circRNAs in MASLD has been recently reviewed elsewhere [15].

Recent estimates of alternative splicing events in the human transcriptome indicate that the vast majority of transcripts, over 95%, are affected by alternative splicing [16]. 

## 3. RNA Splicing: Mechanisms and Regulation

RNA splicing is carried out by the spliceosome, a highly adaptable molecular complex made up of small nuclear RNAs (snRNAs) and various associated proteins [17]. The spliceosome recognizes specific *cis*-regulatory sequences at the exon–intron boundaries, including the 5′ splice site, the branch point, the polypyrimidine tract, and the 3′ splice site. The accurate recognition and excision of intronic sequences are critical for the generation of functional mRNA and, consequently, for the proper expression of genes.

**Figure 2:**
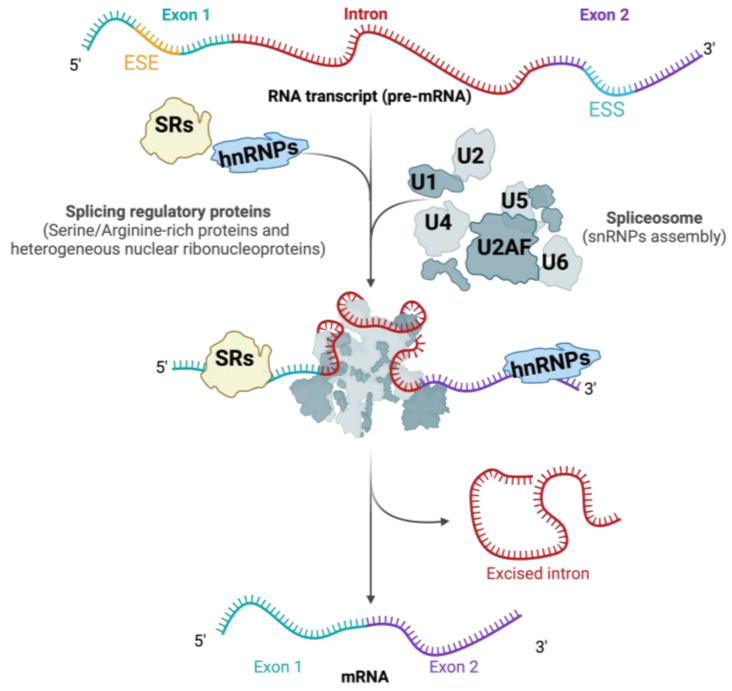
Splicing and spliceosome assembly. Pre-mRNA splicing, facilitated by the spliceosome, involves the stepwise binding and release of snRNPs along with various protein regulators, including SRs (serine-/arginine-rich proteins) and hnRNPs (heterogeneous nuclear ribonucleoproteins). ESE refers to Exonic Splicing Enhancer, while ESS stands for Eonic Splicing Silencer. The figure was created using BioRender (www.biorender.com, accessed on 4 September 2024).

The assembly of the spliceosome initiates when the U1 snRNP identifies the 5′ splice site and the U2 snRNP recognizes the branch point. Subsequently, U4, U5, and U6 snRNPs are assembled to create the fully functional spliceosome [18] (Figure 2). The precise regulation of this process is crucial, as errors in splicing can result in aberrant mRNAs, which in turn may result in the formation of dysfunctional proteins. Splicing dysregulation refers to the abnormal regulation of the splicing process, which can result in aberrant splicing, also known as mis-splicing.

### 3.1. Cis-Regulatory Sequences in RNA Splicing

*Cis*-regulatory sequences play a crucial role in determining the splicing pattern of pre-mRNA. These sequences encompass the 5′ and 3′ splice sites, the branch point, and the polypyrimidine tract. Additionally, exonic and intronic splicing enhancers (ESEs and ISEs) and silencers (ESSs and ISSs) affect the selection or omission of particular exons [19]. The recognition of these sequences by the spliceosome and associated splicing factors ensures the fidelity of the splicing process.

### 3.2. Trans-Acting Splicing Regulatory Proteins

Splicing regulatory proteins are proteins, known also as *trans*-acting splicing factors, that interact with *cis*-regulatory sequences to modulate splicing. The main types of splicing regulatory proteins include serine-/arginine-rich (SR) proteins and heterogeneous nuclear ribonucleoproteins (hnRNPs) [20]. The balance between these splicing factors determines the splicing outcome and, consequently, the diversity of the proteome. Additionally, splicing regulation is affected by other RNA-binding proteins and spliceosome-associated proteins. The interplay between splicing factors and the splicing machinery determines the final mRNA product.

#### 3.2.1. The Role of Serine-/Arginine-Rich (SR) Proteins

SR proteins, characterized by their arginine- and serine-rich (RS) domains, primarily function as splicing activators [18]. They play a critical role in regulating both constitutive and alternative splicing by interacting with splicing enhancer elements and promoting exon inclusion through interactions with other spliceosomal components. These proteins are distinguished by RNA recognition motifs (RRMs) and a C-terminal domain rich in serine and arginine residues [21]. In humans, the core group of SR proteins involved in splicing regulation and various cellular processes includes SRSF1 (also known as ASF/SF2), SRSF2 (SC35), SRSF3 (SRp20), SRSF4 (SRp75), SRSF5 (SRp40), SRSF6 (SRp55), SRSF7 (9G8), SRSF9 (SRp30c), SRSF10 (SRp38), SRSF11, and SRSF12. Several of these proteins have specific functions in the liver.

SRSF1 is pivotal in regulating numerous alternative splicing events. By binding to exonic splicing enhancers (ESEs) and facilitating the recruitment of U1 snRNP to the 5′ splice site [22], SRSF1 promotes exon inclusion. Dysregulation of SRSF1 has been linked to the development of multiple diseases, including cancer and neurological disorders [23,24]. In hepatocellular carcinoma (HCC), SRSF1 promotes tumor infiltration and subsequent metastasis by regulating the alternative splicing of *SRA1* [25].

SRSF2 is essential for splicing genes involved in the regulation of the cell cycle, apoptosis, and stress-responsive alternative splicing events in the liver [26]. It also influences the splicing of genes associated with the proliferation of hepatocytes and the regeneration of liver tissue [27]. Mutations in SRSF2 are associated with various cancers [28], and elevated expression of SRSF2 contributes to the advancement of HCC and its unfavorable prognosis in patients [29].

SRSF3 plays a crucial role in regulating the splicing of genes essential for hepatocyte differentiation and metabolic functions. In mice, the knockout of SRSF3 disrupts the splicing of key regulators involved in glucose and lipid metabolism, including *Dhcr7*, *Ern1*, *Hmgcs1*, *Hnf1α*, *Dhcr7*, and *Scap*, which leads to compromised liver development and function [30].

SRSF5 is implicated in liver cell proliferation during development and liver regeneration [31]. It plays a crucial role in ensuring correct splicing during these processes.

The abundance of SRSF6 is linked with cancer [32], diabetes [33], and liver disease [34]. SRSF6 has been reported to promote the alternative splicing of several mitochondria-associated genes, including *Polg2*, *Nudt13*, *Nme4*, *RnaseI*, and *Guf1*, in both a mouse model of fatty liver disease and in patients with hepatitis [34], indicating its significant role in liver health.

SRSF10 regulates stress-responsive genes [35] and is associated with reduced survival outcomes in patients with liver disease (http://www.proteinatlas.org, accessed on 4 September 2024). *Srsf10* knockout mice exhibit defective liver development [36] and altered gluconeogenesis through *PGC1α* splicing [37], emphasizing its importance in liver metabolism and development.

SFRS10 (official gene name, *TRA2B*) is a member of the SR-like protein family, which plays a key role in the regulation of RNA splicing [38]. Expression of *TRA2B*, a known modulator of *LPIN1* splicing, was shown to be reduced in the livers of obese humans and contributed to increased liver lipogenesis and accumulation of hepatic fat [39].

In conclusion, SR proteins are crucial regulators of gene splicing in the liver, influencing various metabolic and disease-related pathways. Their roles in cell cycle regulation, hepatocyte differentiation, metabolic processes, and stress responses underscore their significance in maintaining liver function and health. Dysregulation of SR proteins can lead to severe liver diseases, including HCC and fatty liver disease, highlighting the potential for therapeutic targets to treat these conditions. Understanding the specific functions of different SR proteins provides valuable insights into liver diseases and potential strategies for treatment.

#### 3.2.2. The Role of hnRNPs

Heterogeneous nuclear ribonucleoproteins (hnRNPs) are a diverse group of RNA-binding proteins that serve essential functions in the regulation of RNA processing, impacting various aspects of RNA metabolism. In the liver, the regulation of gene splicing by hnRNPs is particularly significant, as it influences liver function, metabolism, and response to disease.

Generally, hnRNPs act as splicing repressors, although some can enhance splicing. They achieve this by binding to exonic or intronic splicing silencers (ESSs or ISSs) and blocking the binding of SR proteins or other components of the spliceosome. The primary hnRNPs involved in splicing regulation include hnRNP A1, hnRNP A2/B1, hnRNP A3, hnRNP C1/C2, hnRNP D (also known as AUF1), hnRNP E1, hnRNP F, hnRNP G, hnRNP H1, hnRNP H2 (hnRNP H’), hnRNP I (PTB), hnRNP K, hnRNP L, hnRNP M, hnRNP Q (SYNCRIP), hnRNP R, and hnRNP U. Several hnRNPs have been identified for their specific roles in the liver.

hnRNP A1 and hnRNP A2/B1, for instance, are known to generally promote exon skipping. They modulate the splicing of genes associated with the metabolism of lipids and insulin regulation signaling, such as pyruvate kinase muscle isozyme (*PKM*) [40], which is crucial for glycolysis and has implications in liver metabolism and HCC. Additionally, hnRNP A1 is involved in the splicing of the insulin receptor gene (*INSR*) [41], which plays a pivotal role in insulin sensitivity and glucose homeostasis in the liver.

Another notable hnRNP is hnRNP C1/C2, which acts as a competitor to the splicing factor U2AF65 at numerous splice sites [42]. This competition affects various splicing events. hnRNP C proteins had been associated with HCC [43], highlighting the importance of hnRNP C1/C2 in liver cancer. 

hnRNP D (AUF1) is critical in the breakdown of LDL receptor (*LDLR*) mRNA in the liver [44], impacting cholesterol metabolism and related liver functions.

Moreover, hnRNP F is engaged in the regulation of splicing of the *INSR* [41] gene, similar to hnRNP A1, indicating a collaborative role in insulin signaling regulation.

hnRNP H1/H2 regulates the splicing of genes involved in fructose metabolism, and their dysregulation is linked to HCC [45], further underscoring the connection between hnRNPs and liver cancer.

Another hnRNP of interest is hnRNP Q (SYNCRIP), which is a negative prognostic indicator for liver cancer [46]. Its presence and activity in the liver can influence the progression and severity of liver cancer, making it a potential target for therapeutic interventions.

In conclusion, hnRNPs are essential regulators of gene splicing in the liver, affecting various metabolic and disease-related pathways. Their roles in lipid metabolism, insulin signaling, cholesterol metabolism, and liver cancer highlight their significance in maintaining liver function and health. Understanding the specific functions of different hnRNPs can provide valuable insights into liver diseases and indicate potential intervention points for therapy.

#### 3.2.3. The Role of Other RNA-Binding Proteins

RNA-binding proteins (RBPs) play pivotal functions in controlling gene expression at the post-transcriptional level, particularly in the splicing of pre-mRNAs. These proteins can influence splicing events by encouraging or suppressing the incorporation of particular exons. Notable examples of RNA-binding proteins that influence splicing include Muscleblind-like (MBNL) proteins (MBNL1, MBNL2, MBNL3), Quaking (QKI) proteins (QKI5, QKI6, QKI7), Nova Neuro-Oncological Ventral Antigen (NOVA) proteins (NOVA1, NOVA2), RNA-binding fox (RBFOX) proteins (RBFOX1, RBFOX2, RBFOX3), and epithelial splicing regulatory proteins (ESRP) (ESRP1, ESRP2). Several of these proteins have specific roles in liver function and disease.

Muscleblind-like (MBNL) proteins, encoded by three genes (*MBNL1*, *MBNL2*, and *MBNL3*), are key regulators of tissue-specific RNA splicing. MBNL3 is notably expressed at high levels in fetal liver and HCC but is absent in normal adult liver [47]. Elevated levels of MBNL3 in HCC are linked to the differential splicing of the long non-coding RNA *PXN-AS1*. Specifically, MBNL3 fosters the incorporation of the exon 4, producing the longer *PXN-AS1-L* isoform, which contrasts with the *PXN-AS1-S* isoform found in normal liver. *PXN-AS1-L* influences the stabilization of *PXN* mRNA, leading to elevated levels of paxillin expression [47]. MBNL1 has been demonstrated to modulate the alternative splicing of the insulin receptor (*INSR*) gene [48] with INSR-B isoform, promoting metabolic functions, including glucose uptake and glycogen synthesis. Dysregulation of MBNL1 can lead to an imbalance in these isoforms, affecting liver metabolism and contributing to metabolic disorders.

Quaking (QKI) regulates alternative pre-mRNA splicing and mRNA stability by binding to QKI response elements (QRE) in target RNAs. This regulatory function of QKI is significant in the progression of various cancers, including HCC [49].

RBFOX2 is predominantly expressed in hepatocytes within the liver. Ablation of hepatocyte-specific *Rbfox2* under a lipogenic diet results in reduced blood cholesterol levels but an increase in intrahepatic cholesterol, bile acids, and other lipids, highlighting RBFOX2’s critical role in regulating lipid distribution. RNA-binding fox-1 homolog 2 (RBFOX2) has been demonstrated to be crucial for regulating alternative splicing in liver genes involved in lipid homeostasis, such as *Scarb1*, *Pla2g6*, *Numb*, *Sec31a*, and *Osbpl9* [50].

Epithelial splicing regulatory proteins (ESRP1 and ESRP2) are specialized RNA-binding proteins that play a crucial role in controlling alternative splicing processes, particularly in epithelial cells. ESRP2 has been suggested as a key hepatocyte factor controlling up to 20% of splice isoforms undergoing dynamic changes throughout postnatal liver development [51]. 

In conclusion, these RNA-binding proteins are essential in controlling the process of alternative splicing, impacting liver function and disease. Their ability to modulate splicing events influences various metabolic pathways and cellular processes, highlighting their importance as potential therapeutic targets for liver-related disorders.

While not always considered “regulatory” in the classical sense, the spliceosome-associated proteins are crucial in the assembly and function of the spliceosome. Some of these proteins have regulatory roles in alternative splicing decisions.

## 4. Metabolic Dysfunction-Associated Steatotic Liver Disease (MASLD)

Metabolic Dysfunction-Associated Steatotic Liver Disease (MASLD), formerly known as Non-Alcoholic Fatty Liver Disease (NAFLD) [52], represents a spectrum of liver conditions that are characterized by excessive fat accumulation in the liver, not due to significant alcohol consumption. MASLD is an umbrella term that covers the spectrum of liver disease stages, from simple steatosis (also known as fatty liver, Non-Alcoholic Fatty Liver, or NAFL) to more severe forms like Metabolic Dysfunction-Associated Steatohepatitis (MASH), formerly referred to as Non-Alcoholic Steatohepatitis (NASH). This condition can progress to liver fibrosis, cirrhosis, which is a significant liver scarring, and even HCC. MASLD is closely linked to metabolic dysfunctions such as obesity, insulin resistance, and dyslipidemia. MASLD has also been linked to atherosclerosis, as well as cardiovascular diseases (CVD) such as coronary heart disease and stroke. Consequently, the presence of MASLD is associated with increased vascular risk and the progression of CVD, one of the leading causes of death globally. The evidence connecting obesity, insulin resistance, type 2 diabetes, MASLD, and CVD, along with the molecular mechanisms underlying these diseases, has been thoroughly reviewed elsewhere [53].

Simple steatosis is characterized by the accumulation of fat within liver cells without the presence of inflammation or fibrosis. This condition is generally considered the earliest and relatively benign stage of MASLD and is often asymptomatic, but in some individuals, it can progress to more severe liver conditions if left unmanaged. MASH is characterized by overaccumulation of hepatic fat and necroinflammation of the liver, with or without fibrosis [54]. The transition from steatosis to MASH is crucial, as MASH can progress to cirrhosis and HCC [55]. The connection between MASH and HCC has been reviewed elsewhere [54].

### Understanding MASLD: Pathogenesis and Clinical Implications

MASLD is a major health concern due to its high prevalence worldwide and its potential to progress to severe liver diseases. The pathogenesis of MASLD is complex and multifactorial, involving interplay between genetic predisposition, environmental factors, and metabolic abnormalities. Insulin resistance, lipid metabolism dysregulation, oxidative stress, inflammation, and fibrosis are central to the development and progression of MASLD. MASLD often develops alongside other conditions, such as obesity, type 2 diabetes (T2D), high blood pressure, or high cholesterol, in most individuals. Amid rising rates of obesity, T2D, and metabolic syndrome, MASLD has become the foremost cause of chronic liver disease worldwide and the primary non-viral cause of liver failure necessitating transplantation [56,57].

One intriguing aspect of MASLD lies in the diverse susceptibility observed across ethnic groups [58,59] and mouse strains [60,61]. While genetic, environmental, and lifestyle factors contribute to this variability in susceptibility, the molecular factors underlying health disparities remain largely unknown, impeding the development of effective diagnoses and treatments applicable to diverse populations. Recent advances in genomic and transcriptomic research have highlighted the significant role of RNA splicing in the regulation of genes implicated in MASLD.

## 5. **The Role of Alternative Splicing in MASLD**

Alternative splicing is essential for regulating numerous biological processes, including metabolism, inflammation, and cell survival, all of which are pertinent to MASLD. While the molecular mechanisms driving the variability in MASLD progression to more severe conditions remain unclear, recent research suggests that alternative splicing plays a role in this process. An integrative systems biology approach that merges differential expression analysis with weighted gene co-expression network analysis (WGCNA) using data from 19 normal, 10 simple steatosis, and 16 MASH patients revealed that RNA splicing may play a significant role in the transition from steatosis to MASH [62]. 

Splicing might also be affected by environmental factors; for example, Correia and colleagues showed that *FXR* splice variants play a key role in regulating hepatic lipid metabolism, with their effects varying depending on the specific isoform. Additionally, the splicing of *FXR* is influenced by factors such as feeding status and physical activity [63]. Splicing of *INSR* has been implicated in MASLD. Administering *INSR-A* but not *INSR-B* using adeno-associated viruses (AAVs) in the liver of a mouse model with diet-induced insulin resistance and obesity resulted in enhanced glucose uptake in both the liver and muscle, leading to improved insulin sensitivity. Furthermore, INSR-A expression alleviated hepatic steatosis by reducing the expression of *Fasn*, *Pgc1a*, *Acaca*, and *Dgat2* while increasing *Scd*-*1* expression [64]. 

Dysregulation of splicing can occur due to mutations in either *cis*-regulatory sequences, *trans*-acting regulatory proteins, or through alterations in the expression or function of splicing factors. However, mutations in *trans*-acting factors are relatively rare, likely because alterations in the fundamental components of the splicing machinery are often more detrimental to cell survival than *cis*-acting mutations, which typically affect the splicing of specific genes [65].

Below, several examples of how splicing regulation is implicated in MASLD are explored. While this review focuses on MASLD, the changes to the splicing landscape in liver cancer, including HCC, have been covered in other studies [45,66], including a recent review discussing the role of splicing in the progression from MASLD to HCC [67].

### 5.1. Cis-Regulatory Sequence Mutations and SNPs in MASLD

MASLD is a complex condition driven by an interplay of metabolic, environmental, genetic, and epigenetic factors, affecting multiple organs through diverse mechanisms [68,69]. Li et al. have highlighted the crucial role of RNA splicing in connecting genetic variations to the development of complex diseases, showing that the contribution of aberrant splicing is comparable to that of expression quantitative trait loci (eQTLs) [4]. Notably, their research demonstrated that polymorphisms affecting splicing (sQTLs) and gene expression (eQTLs) function independently, with the majority of sQTLs having no impact on overall gene expression levels (measured as the sum of all transcripts) [4].

Despite numerous candidate gene studies, only a few loci have been identified and validated as being associated with the risk or progression of MASLD [70]. Among these, the single-nucleotide polymorphism (SNP) rs72613567, which influences the splicing of the *HSD17B13* gene, has gained recognition for its protective effect against MASLD [71]. This SNP inserts TA nucleotides, leading to premature termination of the HSD17B13 hepatic lipid-droplet protein, resulting in a truncated, non-functional protein [72]. While this variant does not affect lipid levels or hepatic steatosis, it significantly lowers the risk of advanced MASLD stages, including inflammation, MASH, and liver fibrosis. Emerging evidence suggests that the protective effects of rs72613567 may be limited to specific demographics, such as women, individuals over 45, those with diabetes or obesity, and carriers of the *PNPLA3* I148M variant [73].

Kruppel-like factor (KLF6) plays a key role in regulating the expression of genes involved in fibrogenesis [74] and is upregulated in the MASH model [75], indicating its potential influence on fibrosis severity in human MASLD. The SNP rs3750861, located in the first intron of the *KLF6* gene, affects its mRNA splicing and has been associated with milder hepatic fibrosis in three different European MASLD cohorts [76]. This SNP creates a new binding site for the splicing factor SRSF5, altering *KLF6* splicing [77]. Liver biopsies from MASLD patients showed a correlation between increased levels of full-length *KLF6* expression and the progression to more advanced stages of the disease, characterized by increased steatosis and fibrosis [76].

Another notable SNP, rs10401969, located in intron 8 of the *SUGP1* gene, has been strongly associated with liver lipid content in patients with obesity and MASLD. This polymorphism regulates the skipping of *SUGP1* exon 8, leading to nonsense-mediated mRNA decay [78]. Furthermore, knocking down *SUGP1* in human hepatoma cell lines has been shown to induce alternative splicing of *HMGCR*, reducing cholesterol synthesis and increasing LDL uptake [78].

### 5.2. Splicing Regulatory Proteins in MASLD

The alterations in the liver splicing have been implicated in the oncogenic transformation of the liver [70]. Pihlajamäki et al. demonstrated that the expression of several genes encoding *trans*-acting factors is decreased in both the liver and skeletal muscle of obese humans [39], suggesting that aberrant splicing in obesity might be a general phenomenon. Recently, it was discovered that the expression of 16 out of 45 tested splicing regulating genes differed significantly between patients with and without hepatic steatosis, and significant differences were observed in the expression of eight spliceosome components (RNU6ATAC, RNU6, SF3B1, RNU2, RNU4ATAC, RBM22, U2AF1, U2AF2) and eight splicing factors (PTBP1, SRRM1, SND1, KHDRSB1, SRSF2, SRSF10, ESRP2, TIA1) [79].

Deficiency of SRSF1 in the liver has been linked to the development of MASH-like pathology and hepatocyte cell death. The absence of SRSF1 leads to the accumulation of RNA-DNA hybrids (R-loops), which induce extensive DNA damage. This damage results in global transcriptional repression, impaired splicing, reduced protein synthesis, and metabolic insufficiency, ultimately leading to liver inflammation, fibrosis, and steatosis [80]. The loss of SRSF1 has been shown to affect the splicing of genes involved in metabolic processes, DNA repair, and chromosomal organization. Specifically, SRSF1 depletion in hepatocytes resulted in the mis-splicing of almost 3000 exons [80].

Similarly, SRSF2 plays a crucial role in liver health, with its absence leading to severe pathology, including ballooned hepatocytes and fibrosis, characteristic of MASH [26]. SRSF2 regulates the splicing of genes involved in autophagy and stress responses; its loss triggers isoform switching and reduced expression of key variants, heightening hepatocyte vulnerability to stress, which in turn leads to cholestasis, ER stress, and oxidative stress [26].

A reduction in SRSF3 expression has been noted in both human and mouse liver tissues affected by MASLD, MASH, and HCC [81,82], leading to altered splicing of SFRS3-regulated genes. The early loss of SRSF3 in liver diseases such as steatosis, MASH, and cirrhosis occurs due to proteasomal degradation driven by lipid-induced oxidative stress through neddylation at lysine 11 [82]. Human samples of MASLD, MASH, and cirrhosis revealed alterations in splicing patterns, including increased inclusion of the profibrogenic EDA exon (exon 33) in the fibronectin 1 (*FN1*) gene, exon 23 in the *MYO1B* gene, as well as exon skipping events in exon 11 of the *INSR* gene and exon 13 of the *SLK* gene [82].

SRSF6 is another SR protein linked to the progression of MASLD, primarily through the DRAK2-SRSF6 pathway. This pathway modulates the alternative splicing of genes critical for mitochondrial function. DRAK2’s interaction with SRSF6 inhibits its phosphorylation, altering its splicing activity. Consequently, this affects the splicing of key mitochondrial-related genes, including *Polg2*, *Rnasel*, *Nme4*, *Nudt13*, and *Guf1*, which are essential for maintaining mitochondrial function [83]. 

Reduced expression of SRSF10 is evident in both human MASLD patients and mouse models fed obesogenic diets. Inactivation of SRSF10 in hepatocytes, in both species, triggers intronic polyadenylation and lowers the expression of crucial metabolic genes, such as *PPARα*. Consistently, liver-specific knockdown of *Srsf10* promotes increased cryptic intronic polyadenylation and reduced *PPARα* expression. These molecular changes exacerbate MASLD progression, leading to increased body weight, insulin resistance, and glucose intolerance, especially under obesogenic conditions [84].

hnRNPU deficiency disrupts chromatin structure, leading to a reprogramming of the liver transcriptome that exacerbates MASH pathogenesis. This disruption enhances the expression of inflammatory genes and increases stress-induced hepatocyte injury, contributing to liver fibrosis and inflammation. Additionally, hnRNPU deficiency impacts the splicing of crucial genes, including those involved in inflammatory signaling pathways. This includes the production of a truncated isoform of the TrkB receptor (TrkB-T1), which further amplifies inflammatory signaling and promotes hepatocyte death [85].

## 6. Therapeutic Approaches

Several approaches have been developed to correct mutation-derived splicing defects by targeting either *cis*-regulatory elements or *trans*-acting splice factors. Among these, splice-modulating antisense oligonucleotides (ASOs) have emerged as a promising therapeutic strategy capable of correcting aberrant splicing caused by mutations, thus restoring normal transcript expression and functional protein production. ASOs are typically 15–30 nucleotide long single-stranded synthetic RNA molecules, which, through binding specifically to its mRNA target, can modulate gene expression either by altering mRNA splicing or by promoting the degradation of the mRNA in question [86]. ASO drugs can inhibit exon inclusion either by masking splicing enhancer *cis*-regulatory sequences (ESE or ISE), thereby preventing the binding of *trans*-acting splicing factors, such as SR proteins. Alternatively, they can bind to splicing silencer *cis*-regulatory sequences (ESS, ISS), promoting exon inclusion by blocking the recruitment of *trans*-acting factors such as hnRNPs. This approach offers a highly personalized method for treating various diseases. Over the past decade, significant progress has been made in the clinical application of ASOs. The United States Food and Drug Administration (US FDA) has approved nine antisense oligonucleotide (ASO) drugs for the treatment of various diseases [87,88,89,90,91,92,93,94,95]. Additionally, the European Medicines Agency (EMA) has accepted volanesorsen to treat familial chylomicronemia syndrome (FCS), hypertriglyceridemia, and familial partial lipodystrophy (FPL) [96]. Interestingly, volanesorsen has demonstrated the ability to reduce hepatic fat fraction (HFF) in three different trials [97], highlighting the therapeutic potential of antisense oligonucleotide (ASO) therapy in treating steatosis and opening new avenues for addressing splicing errors in MASLD. While ASOs have shown clinical efficacy, their use is limited, they require intensive therapeutic protocols, including frequent hospital-administered injections, and they come with significant costs.

Recently, the use of small organic molecules to modulate RNA splicing has gained recognition. These small molecules can target various elements involved in the splicing process, ranging from components of the spliceosome to different *trans*-acting splicing factors to RNA targets. For instance, pladienolide-B has demonstrated significant potential in suppressing the growth of HCC cells in both in vitro experiments and xenograft models. Its mechanism of action involves targeting the SF3B complex, leading to the regulation of apoptosis-related proteins like BCL-197 [98]. A key advantage of this approach is that these small organic molecules can be administered orally, removing the necessity for treatment in specialized clinical environments.

In MASLD, aberrant splicing is regulated by SRs and hnRNPs, which in turn are heavily regulated by phosphorylation, by protein kinases from the CLK and SRPK families, as well as kinases activated through various signaling pathways, including Akt, PI3K, and MAPK [99]. Targeting *trans*-acting splicing factors using new small compounds, creates another promising therapeutic approach for the treatment of mis-splicing. However, this approach affects the activity of multiple proteins in multiple different cells and thus lacks specificity. 

In the context of severe obesity, individuals show diverse patterns of liver disease progression: some remain free of MASLD, others develop only benign steatosis, and a subset advance to more severe conditions such as MASH, cirrhosis, and even HCC. This variability prompts the question of whether splicing alterations, which are known to drive disease progression, might serve as predictive biomarkers for the disease’s trajectory. Recent studies suggest that splicing factors implicated in HCC may hold a prognostic value, indicating the potential for splicing alterations to predict liver disease outcomes [98].

## 7. Conclusions

Alternative splicing is a fundamental regulatory mechanism that significantly contributes to the complexity of gene expression and proteomic diversity in liver cells. Dysregulation of this process, whether due to mutations in *cis*-regulatory sequences or alterations in *trans*-acting splicing factor activity, can have profound effects on the pathogenesis of MASLD. A deeper understanding of the mechanisms governing splicing in MASLD offers critical insights into the disease’s etiology and highlights potential therapeutic targets.

Looking ahead, investigating alternative splicing in MASLD presents exciting opportunities for both foundational research and clinical innovation. Future studies should aim to uncover the specific splicing events and regulatory networks that drive the progression from simple steatosis to more advanced stages like MASH, cirrhosis, and HCC. Key questions remain as to whether sex and ethnic differences in alternative splicing landscapes play a role in susceptibility to these conditions. In the long term, splicing–modulating therapies—such as ASOs and small molecules—show great promise for personalized treatment strategies. These therapies could be designed to correct patient-specific splicing defects, potentially halting or even reversing disease progression. Additionally, integrating splicing biomarkers into diagnostic frameworks could improve early detection and help identify individuals at higher risk for disease progression. Ultimately, deepening our understanding of RNA splicing in liver diseases will be crucial for developing more effective treatments for MASLD and other related metabolic disorders.

## Data Availability

Data sharing is not applicable.
